# A Novel *In Vivo* Model of Focal Light Emitting Diode-Induced Cone-Photoreceptor Phototoxicity: Neuroprotection Afforded by Brimonidine, BDNF, PEDF or bFGF

**DOI:** 10.1371/journal.pone.0113798

**Published:** 2014-12-02

**Authors:** Arturo Ortín-Martínez, Francisco Javier Valiente-Soriano, Diego García-Ayuso, Luis Alarcón-Martínez, Manuel Jiménez-López, José Manuel Bernal-Garro, Leticia Nieto-López, Francisco Manuel Nadal-Nicolás, María Paz Villegas-Pérez, Larry A. Wheeler, Manuel Vidal-Sanz

**Affiliations:** 1 Departamento de Oftalmología, Facultad de Medicina, Universidad de Murcia, and Instituto Murciano de Investigación Biosanitaria Virgen de la Arrixaca (IMIB-Arrixaca), Murcia, Spain; 2 Zeteo Drug Discovery LLC, Irvine, California, United States of America; Dalhousie University, Canada

## Abstract

We have investigated the effects of light-emitting diode (LED)-induced phototoxicity (LIP) on cone-photoreceptors and their protection with brimonidine (BMD), brain-derived neurotrophic factor (BDNF), pigment epithelium-derived factor (PEDF), ciliary neurotrophic factor (CNTF) or basic fibroblast growth factor (bFGF). In anesthetized, dark adapted, adult albino rats a blue (400 nm) LED was placed perpendicular to the cornea (10 sec, 200 lux) and the effects were investigated using Spectral Domain Optical Coherence Tomography (SD-OCT) and/or analysing the retina in oriented cross-sections or wholemounts immune-labelled for L- and S-opsin and counterstained with the nuclear stain DAPI. The effects of topical BMD (1%) or, intravitreally injected BDNF (5 µg), PEDF (2 µg), CNTF (0.4 µg) or bFGF (1 µg) after LIP were examined on wholemounts at 7 days. SD-OCT showed damage in a circular region of the superotemporal retina, whose diameter varied from 1,842.4±84.5 µm (at 24 hours) to 1,407.7±52.8 µm (at 7 days). This region had a progressive thickness diminution from 183.4±5 µm (at 12 h) to 114.6±6 µm (at 7 d). Oriented cross-sections showed within the light-damaged region of the retina massive loss of rods and cone-photoreceptors. Wholemounts documented a circular region containing lower numbers of L- and S-cones. Within a circular area (1 mm or 1.3 mm radius, respectively) in the left and in its corresponding region of the contralateral-fellow-retina, total L- or S-cones were 7,118±842 or 661±125 for the LED exposed retinas (n = 7) and 14,040±1,860 or 2,255±193 for the fellow retinas (n = 7), respectively. BMD, BDNF, PEDF and bFGF but not CNTF showed significant neuroprotective effects on L- or S-cones. We conclude that LIP results in rod and cone-photoreceptor loss, and is a reliable, quantifiable model to study cone-photoreceptor degeneration. Intravitreal BDNF, PEDF or bFGF, or topical BMD afford significant cone neuroprotection in this model.

## Introduction

In mammals nigthtlight (scotopic) vision is carried out by rod-photoreceptors, while cone-photoreceptors are responsible for daylight (photopic) vision and colour discrimination. In nonprimate mammals, photopic vision is achieved by two types of cones, each carrying an opsin responsible for detection of short (S-cones) and medium to long (L-cones) wave lengths, respectively. S-cones express the ultraviolet sensitive or SWS1 opsin and L-cones express the LWS opsin which detects green light and is referred as the L-opsin. These opsins, expressed in the outer segment, may be identified with immunohistochemistry and used as reliable markers to identify rodent cone-photoreceptors [1]–[3]. The albino rat retina, as in most mammals, is an L-cone-dominated retina with an L-to-S-cone mean ratio of 6∶1 [1]. Although the rat retina does not have a proper macula, it has a visual streak with highest concentrations of RGCs and L-cones, but mostly devoid of S-cones, in an horizontal region along the dorsal retina [1],[2],[4]–[6].

Retinal exposure to either excessive or short-wavelength light may result in damage to photoreceptors [7] or to their functional counterpart [8], the retinal pigment epithelial cells (RPE) [9]. Both types of cells contain light-absorbing pigments, the photoreceptors contain the opsins responsible for phototransduction while RPE contain pigments (e.g., melanin and lipofuscin) that absorb scattered light to improve optical quality [8]. Photochemical damage [10], [11] may involve the formation of free radicals and thus oxidative stress following excessive photoreceptor photopigment activation [7] or the effect of short-wavelength linked to chemical changes in lipofuscin [9]. Light-induced retinal damage has been used as a model for animal and human photoreceptor degenerations [12]–[14], including inherited animal models of retinal degeneration and atrophic age-related macular degeneration (AMD) [15]. Indeed, excessive light is a known risk factor for AMD [16], [17], the most common cause of vision loss in human over the age of 65 [18], and in the pathogenesis of this disease it has been postulated that photochemical damage might be mediated by oxidative stress [19].

Numerous studies have reported the effects of light-induced phototoxicity on photoreceptors, both in rats and mice, as well as the effects of several neuroprotective agents [11], [20]. Indeed, several studies have reported the neuroprotective effects of alpha-2 selective agonist [21] as wells as of trophic factors including BDNF, CNTF, PEDF and bFGF [22]–[35], against light-induced photoreceptor cell loss. Most of the above mentioned studies however, have reported the response of photoreceptors as a whole without making distinctions between different types of photoreceptors and to date, there is little to none information regarding the specific response of L- or S-cone-photoreceptors and their possible rescue in *in vivo* murine models of phototoxicity.

The rat retina provides an excellent model to study short and long-term neuronal responses against a variety of injuries or diseases, including phototoxic-induced [12]–[14], [36] or inherited models [37]–[43] of photoreceptor degeneration. In our laboratory, we have recently developed automated routines to identify, count and map the topography of the entire population of adult rodent retinal ganglion cells (RGCs) both in control [4]–[6], [44]–[46] or injured retinas [13], [14], [41], [47]–[56], as well as of S- and L-cones, both in control [1], [2] or injured retinas [3]. Optic coherence tomography (OCT) is a technique used in ophthalmology for more than 20 years to study the retina *in vivo*. More recently, Spectral Domain (SD) OCT resolution has been introduced in clinics and also in animal studies [57]–[63].

The aim of this study is to characterize an *in vivo* model of focal retinal phototoxicity-induced cone-photoreceptor degeneration in adult albino rats that would allow for longitudinal *in vivo* and *ex vivo* assessment. Thus, one primer objective is to establish quantitatively the pattern of *cone photoreceptor* cell loss that follows light-induced phototoxicity of the retina. Rather than simply sampling areas of the retina, as has been the rule to obtain quantitative and qualitative data for previous retinal light-induced damage studies, the state of the art technologies employed in the present manuscript (i.e. automatic quantification of whole L- and S-cone population in retinal wholemounts and detailed topological representation of injured/remaining cone-photoreceptors within the retina in color-coded isodensity maps) allows specific investigation of the entire populations of L- and S-cones. This becomes even more relevant if one takes into account that, on average, approximately 99% of the photoreceptors in the rat are rods while cones account for only 1% [64], [65]. Thus, the response of rat cone-photoreceptors to this type of lesion, even though probably linked to the fate of rods, has been rarely studied specifically. Moreover, we succeed in directing the damage specifically to the retinal region that contains the peak L-cone and retinal ganglion cell densities, and thus may be used to study cone-photoreceptor injury. A second important objective is to explore the neuroprotective effects of BMD (topically applied) or BDNF, bFGF, PEDF or CNTF (injected intravitreally), on cone survival after phototoxicity.

Here we present a new reproducible and quantifiable model of focal cone degeneration induced in the temporal tip of the rat visual streak by blue-Light Emitting Diode (LED) photoexposition. Moreover, we document neuroprotection with several neurotrophic factors and topical BMD. It is anticipated that these data could serve as a baseline for further studies aimed at investigating cone-photoreceptor neuroprotection. Short accounts were reported [66], [67].

## Material and Methods

### Animal handling

This study was carried out in strict accordance with the recommendations in the Guide for the Care and Use of Laboratory Animals of the Association for Research in Vision and Ophthalmology (ARVO) and the European Union guidelines for the use of animals in research, and all used protocols were approved by the Ethical and Animal Studies Committee of the University of Murcia. Adult female albino Sprague-Dawley (SD) rats (180–230 g) obtained from Charles River Laboratories (L'Arbresle, France) were housed at the University of Murcia animal facilities, in temperature and light controlled rooms (12 h light/dark cycle) with food and water “ad libitum”. Light intensity within the cages ranged from 5 to 30 lux. Surgeries and animal manipulations were performed under general anaesthesia [intraperitoneal (ip) injection of xylazine (10 mg/kg body weight (bw), Rompun; Bayer, Kiel, Germany) and ketamine (60 mg/kg bw, Imalgene; Merial Laboratorios, Barcelona, Spain)], and all efforts were made to minimize suffering. Oral analgesia was provided from the day of experimental manipulation to the day of sacrifice (Buprex, Bupernorphine 0.3 mg/mL, Shering-Plough, Madrid, Spain, was prepared in gelatin strawberry-flavoured at 0.5 mg/kg). During recovery topical ointment containin Tobramycin (Tobrex Alcon-Cusí, S.A., El Masnou, Barcelona, Spain) was applied to prevent corneal dessication. Animals were sacrificed with an ip overdose of pentobarbital (Dolethal, Vetoquinol, Especialidades Veterinarias, S.A., Alcobendas, Madrid, Spain). In all rats the left eyes were used as experimental and the contralateral fellow-right eyes served as controls.

### Experimental design and Animal groups

**Figure 1:**
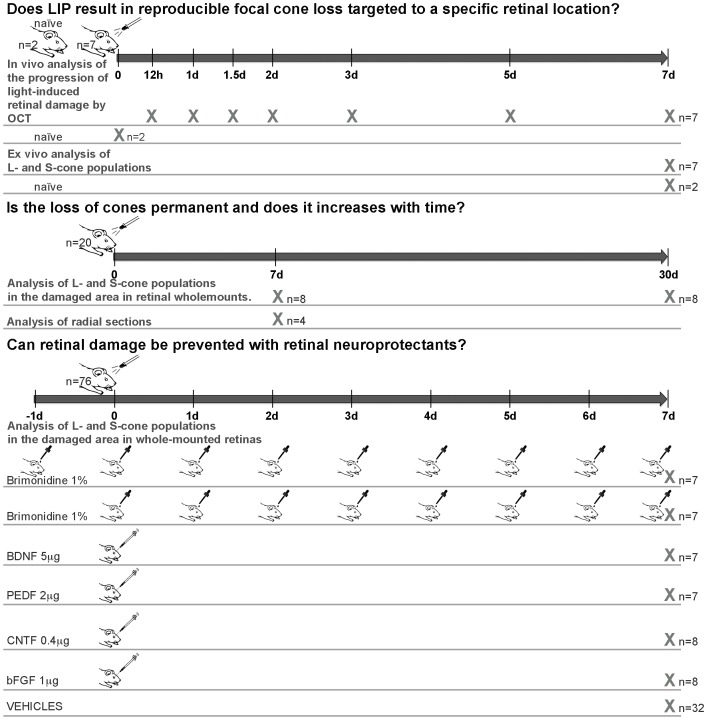
Experimental design. Outline of the time frame, animals and techniques employed to investigate the effects of blue-light emitting diode-induced phototoxicity (LIP) in the adult albino rat retina. Preliminary experiments served to determine optimal parameters to obtain consistent damage to the outer retinal layers. Because consistent results were obtained with intensities of 200 lux and time exposure intervals of 10 sec, these parameters were used thereafter. 1. To investigate if LIP results in reproducible focal loss of L- and S-cones in specific targeted retinal locations we have analyzed *in vivo* with SD-OCT the progression of LIP retinal damage in rats without further treatment (n = 7) and in naïve untouched rats (n = 2), as well as *ex vivo* the populations of L- and S-cones in these retinas. 2. To determine if the loss of cones is permanent and if the area of the lesion increases with time we have; i) analyzed at 7 days after LIP the retinas of a group of rats in oriented radial parasagittal sections obtained in a freezing microtome (n = 4), and; ii) compared two groups of rats examined 7 (n = 8) or 30 days (n = 8) after LIP. 3. Can LIP be prevented with retinal neuroprotectants? To study neuroprotection in this model several groups of rats were treated with: i) brimonidine pre-LIP (n = 7); ii) brimonidine post-LIP (n = 7); iii) brain-derived neurotrophic factor (BDNF) (n = 7); iv) pigment epithelium-derived factor (PEDF) (n = 7); v) ciliary neurotrophic factor (CNTF) (n = 8); vi) basic-fibroblast growth factor (bFGF) (n = 8), and their corresponding control vehicle groups; vii) saline pre-LIP (n = 7); viii) saline post-LIP (n = 8); ix) phosphate buffered saline (PBS) (n = 9), and; x) Tris-Cl 2 mM pH 7,6 (n = 8).

The study addressed the following questions regarding blue-light emitting diode (LED)-induced phototoxicity (**LIP**): 1) Does LIP result in reproducible focal loss of L- and S-cones and, can the lesion be targeted to a specific retinal location? 2) Is the loss of cones permanent or does it progress with time? And, 3) Can retinal damage be prevented with retinal neuroprotective substances? (**Fig. 1**).

### Light emitting diode induced-phototoxicity

Animals were dark adapted overnight (at least 12 h; [68]) and subsequent manipulations were conducted under dim red light (λ>600 nm). One hour prior to LIP, rats were anaesthetized and pupil mydriasis was induced in the left eye with one drop of tropicamide (Tropicamida 1%; Alcon-Cusí, S.A., El Masnou, Barcelona, Spain). LIP was performed in the left eye between 10–12 am to avoid variations in the amount of retinal toxicity related to the time of light exposure [11], [68],[69]. The rat's head was placed horizontal on a head-holder and the left eye was exposed to blue-light (400 nm) by a light emitting diode (LED) (emission spectrum 390–410; catalogue number 454–4405; Kingbright Elec. Co., Taipei, Taiwan) connected to a computer to control for duration of exposure (10 secs) and intensity of light (200 lux; lux intensity was measured with a luxometer (light meter TES-1330; TES Electrical Electronic Corp., Taipei, Taiwan) placed 1 mm below and perpendicular to the LED). The LED was held by a micromanipulator and placed 1 mm above and perpendicular to the corneal apex of the left eye. Tsukahara and colleagues [70] have estimated the transmittance of the ocular media (cornea and crystalline lens) at different wavelengths in rats, and found that for blue-light (400 nm) the energy that reaches the retina is ≈78%. Thus we assume that under our experimental conditions approximately 80% of the energy provided by the LED reaches the retina.

### Spectral Domain Optical Coherence Tomography (SD-OCT)

In naïve (n = 2) or light exposed (n = 7) anaesthetized rats, the left pupil was dilated and a custom-made contact lens was placed on the cornea. Retinal OCT was carried out with a SD-OCT device (Spectralis; Heidelberg Engineering, Heidelberg, Germany). To adapt for the optical qualities of the rat eye, a commercially available 78-D double aspheric fundus lens (Volk Optical, Inc., Mentor, OH) was mounted directly in front of the camera unit. Imaging was performed with a proprietary software package (Eye Explorer, version 3.2.1.0; Heidelberg Engineering). Length of the reference pathway was adjusted manually according to manufacturer's instructions. To analyse the progression of the damage caused by the LIP, the experimental eyes were examined (scan angle 55°) 12 h, 1, 1.5, 2, 3, 5 and 7 days after LIP. At the same times of study, using scan OCT sections formed by 6 diagonal in star shape with its centre located in the centre of the lesion (scan angle 30°), we measured the length of the lesion and the retinal thickness in the centre of the lesion. These animals were sacrificed 7 days after LIP and their retinas prepared as wholemounts and processed for immunohistochemistry (see below).

### Neuroprotective compounds (Doses, frequency and routes)

Two different routes of administration and five different neuroprotective compounds were tested in this work. An alpha-2-adrenergic agonist, 1% BMD in 0.9%NaCl (Allergan Inc. Irvine, CA, USA) was administered topically (two 5 µl drops three times a day) starting the day before LIP (pre-LIP group; n = 7) or immediately after (post-LIP group; n = 7). Right after LIP, 5 µl were injected intravitreally [71], [72] containing 5 µg of BDNF (Preprotech, London, UK) (n = 7), 2 µg PEDF (Preprotech, London, UK) (n = 7) or 0.4 µg of CNTF (R&D Systems; Vitro S.A. Madrid, Spain) (n = 8), all diluted in phosphate buffered saline (PBS), or 1 µg of bFGF (Preprotech, London, UK) (n = 8) diluted in Tris-Cl 2 mM pH 7.6. Additional groups of control rats were treated with corresponding vehicle solutions.

### Tissue processing

After deep anaesthesia, the superior pole of the eye was marked with a silk 6/0 suture to maintain proper orientation [1], [3]–[5], [73], then rats were perfused through the heart with saline and 4% paraformaldehyde in 0.1 M PB, both eyes enucleated and further processed to obtain wholemounts or cross-sections, and the L- and S-cones were immunodetected by their specific opsin expression as described in detail [1]. In brief, retinal wholemounts or cross-sections were incubated in 1∶1000 goat anti-OPN1SW (N-20) (Santa Cruz Biotechnology, Heidelberg, Germany. Lot# L1906, sc-14363) and in 1∶1200 rabbit anti-opsin red/green (Chemicon-Milipore Iberica, Madrid, Spain. Lot # 2210352, AB 5405) and these were visualized with secondaries 1∶500 Alexa Fluor-488 donkey anti-goat IgG (H+L) (Molecular Probes-Invitrogen, Barcelona, Spain. Lot # 1182671, A11055), or 1∶500 Alexa Fluor-594 donkey anti-rabbit IgG (H+L) (Molecular Probes-Invitrogen, Barcelona, Spain. Lot # 1107500, A21207), respectively. All antibodies were diluted in phosphate buffered saline (PBS) containing 2% Triton X-100 (Sigma-Aldrich, Madrid, Spain).

### Retinal Analysis

We analysed a group of retinas 7 d after LIP in radial-sections to determine whether the lack of opsin-immunoreactivity reflected opsin downregulation or cone-photoreceptor loss, and to investigate if LIP in these retinas had also affected rods. Oriented 14 µm thick cross-sections cut in the parasagittal plane were obtained in a freezing microtome, doubly immunoreacted for L- and S-opsin and also stained for DAPI [1]. Retinal wholemounts or cross-sections (left and right eyes) were examined for L-opsin (detected with Alexa Fluor-568, red signal) and S-opsin (detected with Alexa Fluor-488, green signal) and photographed with a microscope (Axioscop 2 Plus; Zeiss) equipped with a digital-high-resolution camera (ProgResTM c10; Jenoptic, Jena, Germany) and a computer-driven motorized stage (ProScanTM H128; Prior Scientific Instruments Ltd., Cambridge, UK) connected to an image analysis system (Image-Pro Plus 5.1 for Windows; Media Cybernetics, Silver Spring, MD) and a microscope controller module (Scope-Pro 5.0 for Windows; Media Cybernetics). Photomontages of wholemounts or cross-sections were constructed from 154 consecutive frames captured on the microscope side by side with no gap or overlap between them. All images were captured at a resolution of 300 dpi. Reconstructed images were further processed with image-editing computer software (Adobe Photoshop CS; ver. 8.0.1; Adobe Systems, Inc., San Jose, CA), when correct orientation of the retina or image coupling was needed. The area of retinal wholemounts was measured on the high-resolution photomontage image of the complete retina, with the software (IPP; Media Cybernetics). The light exposed retinas showed a small region of an approximate circular shape with diminished numbers of L- and S-cones with its centre consistently located at approximately 3,4 mm from the optic disc in the superotemporal quadrant. This region was delineated and measured (IPP; Media Cybernetics).

### Counts of L- or S-cones in two predetermined fixed-size c*ircular areas* (PCA) and isodensity maps

**Figure 2:**
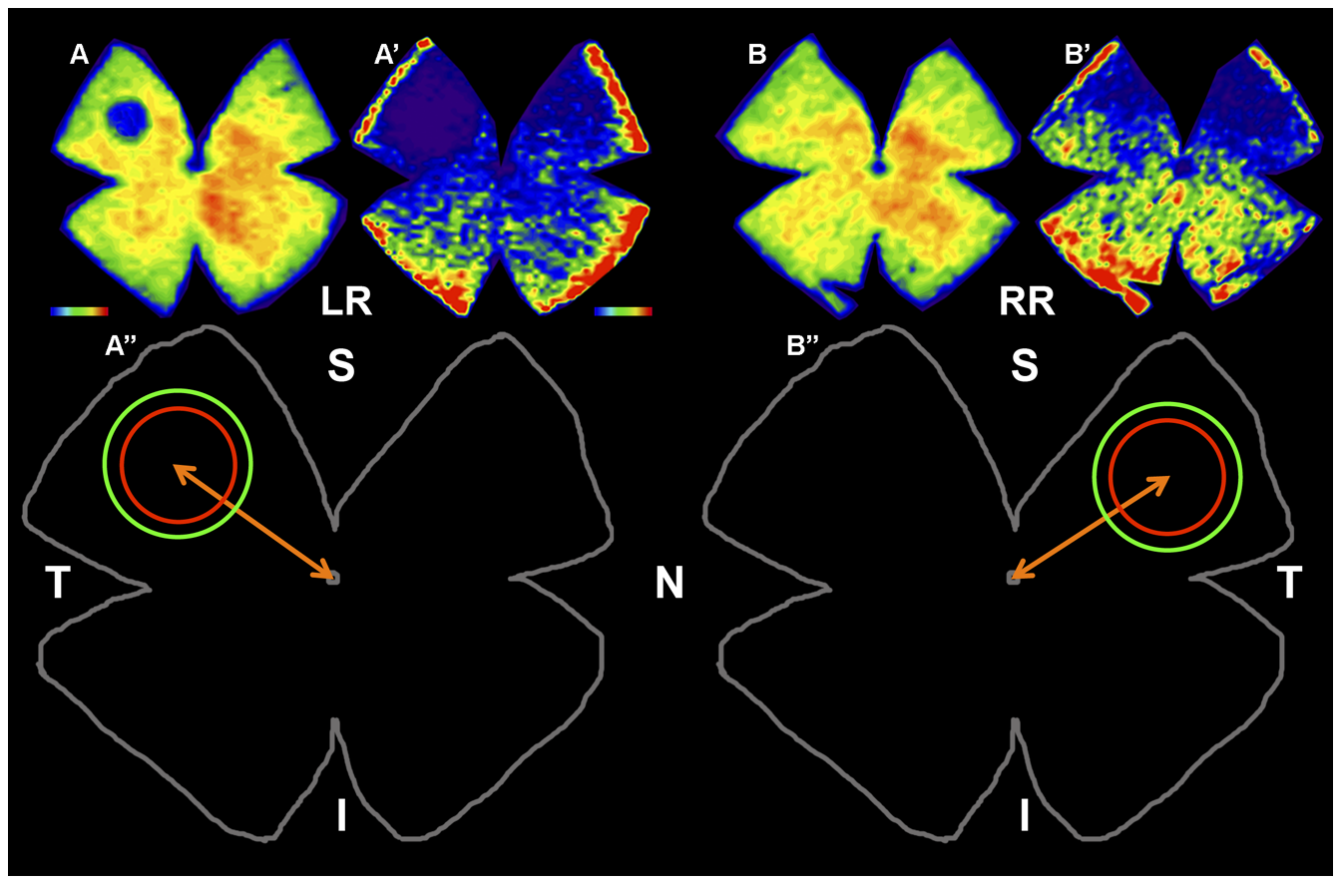
Predetermined fixed-size circular areas (PCA) to count L- or S-cones in left experimental (LR) and right (RR) retinas. A,A′,B,B′. Isodensity maps of a representative left retina (LR) exposed to blue-light emitting diode induced phototoxicity (LIP) in the left eye and its fellow right retina (RR). Seven days after LIP the retinas were dissected as flattened wholemounts and processed for L- and S-opsin immunohistofluorescence. Isodensity maps were represented as a filled contour plot generated by assigning to each one of the subdivisions of each individual frame a color code according to its L- or S-cone density value within a color-scale range from 0 (purple) to 6,500 or higher L-cones/mm^2^ (red), or from 0 (purple) to 1,300 or higher S-cones/mm^2^ (red). Note the presence in the light exposed left retina (LR) of a small circular region of decreased cone density located in the superotemporal quadrant, which is greater for the S- (A′) than for the L-opsin immunoreactivity (A), and the absence of a noticeable lesion in the fellow right retinas (B,B′). A″, B″. Outlines of the retinas shown above to illustrate that L- or S-cone immunopositive outer segments were counted within a predetermined fixed-size circular area (PCA) centred on the lesion with a radius of 1 mm for L- (red) and 1.3 mm for S-cones (green), in the left retina (A″) and in its corresponding location on the right retina (B″). S, superior; T, temporal; I, inferior; N, nasal. Bar =  1 µm.

Total numbers of remaining L- and S-cones were counted automatically on retinal wholemounts using recently developed routines that count labelled cone outer-segments [1]–[3], [42], and the data were translated into isodensity maps (Sigmaplot 9.0, Systat Software Inc., Richmond, CA) as reported [1]–[3] to allow the visualization of detailed topological distribution. The light exposed retinas showed a damaged region of circular shape, with diminished densities of L- and S-cones and an almost absence of these in its centre. Thus, an additional macro was designed to count remaining L- or S-opsin immunoreactive cones within *predetermined fixed-size circular areas* (PCA) of the retina that comprised the light-damaged region, which was larger for S- than for L-cones, and thus PCA were larger for S- (radius of 1.3 mm) than for L- (radius of 1 mm) cones (**Fig. 2**). For each experimental animal, these counts were obtained from the left retina and from a corresponding region in their right-fellow retina (**Fig. 2**). An additional macro enabled representation of the number of L- or S-opsin immunoreactive cone outer-segments within these PCA in both retinas.

### Statistical Analysis

Statistical analysis were performed with SigmaStat for Windows version 3.11 (Systat Sofware, Inc., Richmond, CA), differences were considered significant when p<0.05 and tests are detailed in Tables.

## Results

### Optimization of LED induced phototoxicity parameters

To develop this *in vivo* model, we had previously analysed numerous different combinations of light intensities and exposure times to produce a small but well demarcated region of retinal damage. Light intensities (100-500 lux) and time-exposures (5 sec-5 minutes) were explored, and these produced small focal lesions of increasing size with increasing intensities, time-exposures or both. In additional experiments, the location of the LED with respect to the main eye axis was modified with a micromanipulator to target the injury to the highest L-cone-density area of the retina located in the superotemporal quadrant. Consistent results were obtained with intensities of 200 lux and time exposure intervals of 10 sec, and thus, these parameters were used thereafter.

### *In vivo* and *ex vivo* analysis of LIP-retinal damage

#### In vivo SD-OCT analysis

**Figure 3:**
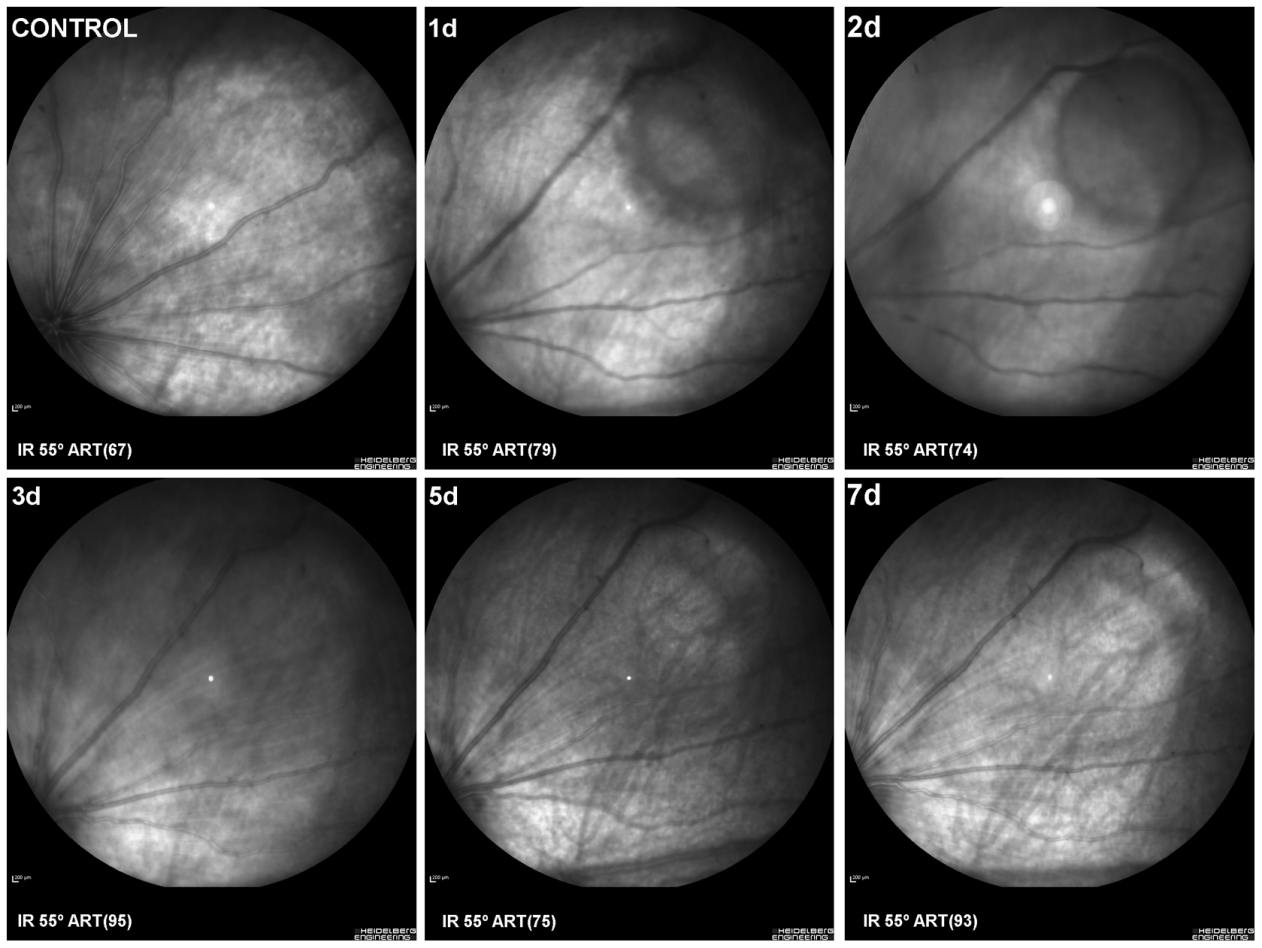
SD-OCT-eye fundus image showing *in vivo* the evolution of the retinal damage. Fundus eyes images of a control naïve retina (Control) and an experimental retina to illustrate the location within the retina with respect to the optic disc as well as the progression of damage throughout the time period of the study, from 1 to 7 days after blue-light emission diode induced phototoxicity. IR: Infrared mode. ART: number of frames averaged.

**Figure 4:**
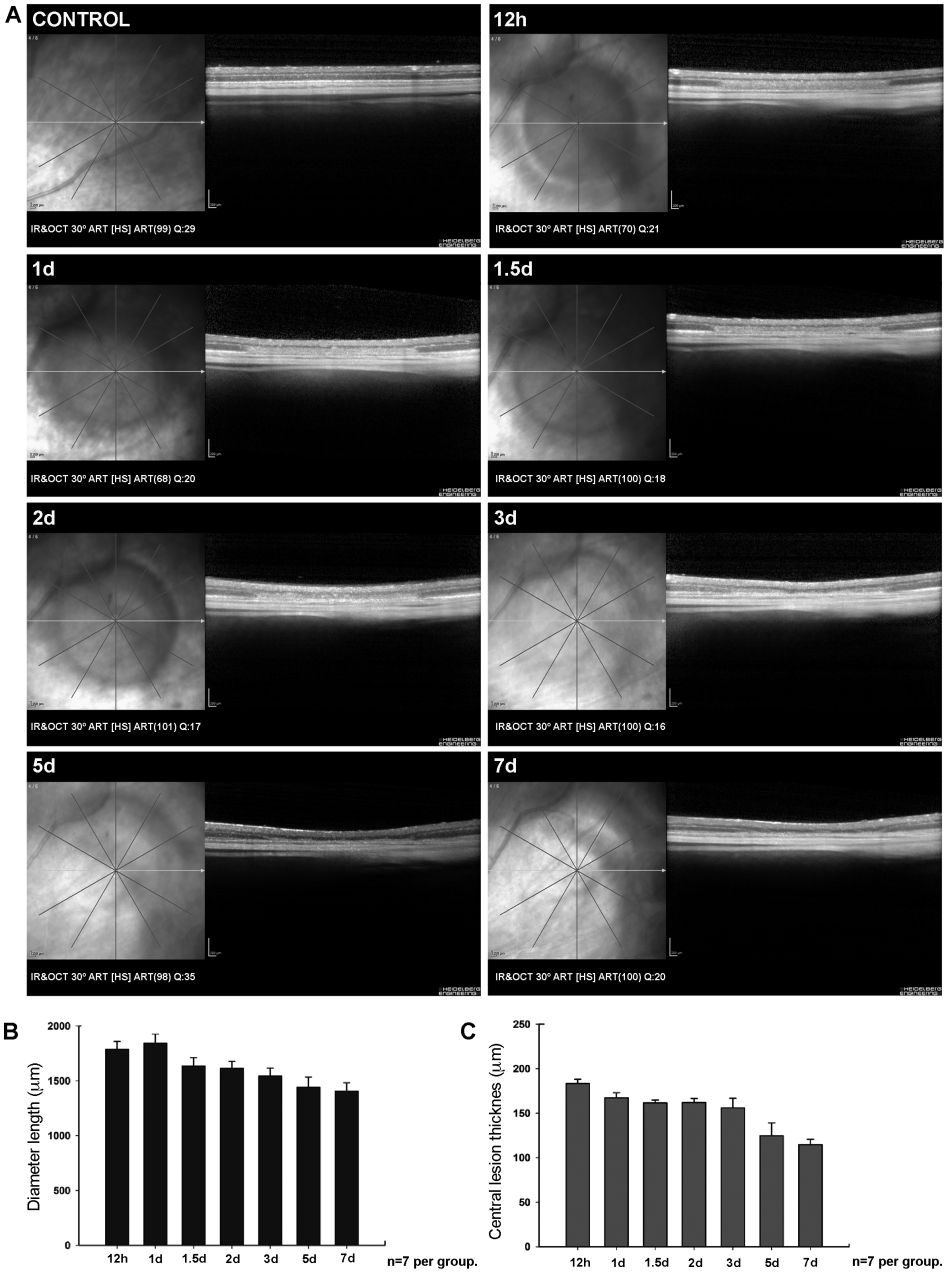
SD-OCT analysis showing the in vivo progression of LIP in one animal. Fundus eyes images and SD-OCT corresponding scans showing a control retina and one experimental eye at increasing survival intervals (12 hours-7days) after blue-light emitting diode induced retinal phototoxicity in a representative left retina (same retina as shown in Fig.3). The histograms show the diameter of the damaged region (B) and the retinal thickness in the centre of the lesion area (C). A. Retinal damage was circumscribed to a circular region of approximately 1.8 mm diameter within the superotemporal quadrant (the region with highest L-cone densities). B,C. Histograms show analysis of diameter (B) and retinal thickness in the central lesion area (C). B. The length of maximal diameter of the lesion decreased from 24 hours (1,842.4±84.5 µm) to 7 days (1,407.7±52.8 µm) after LIP. C. There was progressive diminution of the retinal thickness in the centre of the damaged region from 12 hours (183.4±5 µm) to 7 days (114.6±6 µm). IR: Infrared mode. OCT: Optical Coherence Tomography mode. HS: High speed. ART: number of frames averaged. Q: quality of image on a scale of 1–50.

LIP was analysed *in vivo* and in a group of control-naïve (n = 2) and experimental (n = 7) rats (**Figs. 3** **–** **4**). The left retinas were imaged *in vivo* using SD-OCT at 12 hours, 1, 1.5, 2, 3, 5 and 7 days after LIP. Retinal damage was circumscribed to a circular region of approximately 1.8 mm diameter. Fundus eyes images (**Fig. 3**) and OCT scans (**Fig. 4**) show the progression of retinal damage at increasing survival-intervals after LIP (**Figs. 3** **–** **4**). There was a progressive diminution of the retinal thickness in the centre of damage from 12 hours (183.4±5 µm) to 7 days (114.6±6 µm). Quantitative analysis indicates that the thinning was mainly due to thickness diminution of the outer nuclear (ONL) and outer segment layers (OSL) of the retina (**Fig. 4**). The maximal diameter of the damaged region decreased from 24 hours (1,842.4±84.5 µm) to 7 days (1,407.7±52.8 µm) after LIP (**Fig. 4**). In naïve rats, the mean retinal thickness was 192.7±5 µm.

#### Ex vivo Immunocytochemical analysis

**Figure 5:**
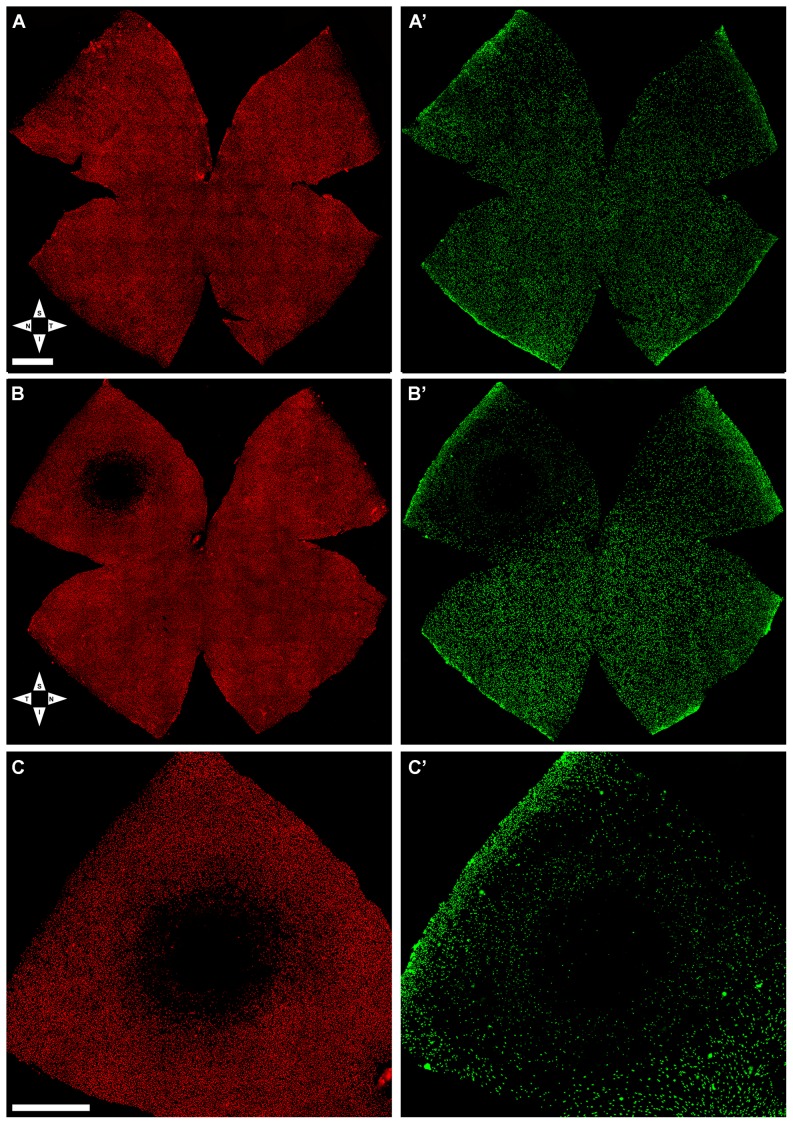
Light-induced focal damage to the cone-photoreceptor population. Wholemount of the fellow-right (unexposed) (A,A′) and the left (light exposed) (B,B′) retinas from a representative rat seven days after blue-light emitting diode induced retinal phototoxicity. L- (A,B,C) and S- (A′,B′,C′) cones were labelled with antibodies against the different opsins. A,A′. In the control retina, L- (A) and S-cones (A′) appear normally distributed throughout the retina. B,B′. The light exposed retina (same retina illustrated in Figs.3–4), shows a small region with reduced densities of L- and S-cones in the superotemporal quadrant at approximately 3.4 mm from the optic disc. Note that the area is larger for the S- than for the L-opsin. C, C′. Details of the superotemporal quadrant of the same retina (shown in B,B′) demonstrating the typical circular damage induced by phototoxicity. S, superior; T, temporal; I, inferior; N, nasal. Bar: 1 mm.

Following last OCT analysis the retinas were processed for fluorescence microscopy. The right-fellow retinas showed highest concentrations of L-cones, and lowest of S-cones, in a horizontal region along the superior naso-temporal axis, approximately 1 mm above the optic disc with maximum values in the superotemporal quadrant, and this is consistent with the normal parameters described for the albino rat retina [1] (**Fig. 5**). In contrast, the light-exposed retinas showed a small circular region with diminished L- and S-opsin immunofluorescence, consistently positioned in the superotemporal quadrant with their centre located at approximately 3.4 mm from the optic disc. When measured, the regions lacking L-opsin immunoreactivity had a mean (±SD) value of 0.68±0.17 mm^2^ (n = 7) with a range between 0.51 and 0.94 mm^2^, whereas for the S-cones the damaged region, which was also of circular shape whit its centre in the same location as for the L-cones, the area was significantly larger with a mean value of 3.57±0.38 mm^2^ (n = 7) and a range between 2.99 and 4.03 mm^2^ (**Table 1,** **Fig. 5**). There were no significant differences in the mean total area of the experimental retinas when compared to their fellow- or to naïve-retinas (**Table 2**).

**Table 1:** Quantitative analysis of LIP retinal damage.

	Total number of cones in retinal wholemounts				Cones in predetermined fixed-size circular areas (PCA)				Area of retina		Area of damaged)		Diameter length of damage (µm)			
Rat	L-cones		S-cones		L-cones		S-cones		(mm^2^)		region (mm^2^		Measured on retinal wholemounts			Measured
	Right retina	Left retina	Right retina	Left retina	Right retina	Left retina	Right retina	Left retina	Right retina	Left retina	L-cones	S-cones	L-cones	S-cones	average	by OCT
C	215,894	200,429	34,892	35,707	14,521	7,025	2,556	625	58.2	55.0	0.57	2.99	952	1,882	1,417	1,390
D	219,235	223,050	41,258	35,057	17,001	7,753	1,998	496	58.3	57.6	0.76	3.92	892	2,105	1,499	1,443
E	206,983	215,943	37,489	28,757	13,986	6,136	2,204	729	56.2	57.2	0.86	3.69	986	2,039	1,513	1,404
F	236,874	193,050	30,980	36,994	11,587	5,897	2,369	642	56.6	58.6	0.94	3.18	1,107	1,701	1,404	1,319
G	238,025	210,199	39,568	29,876	14,789	7,249	2,179	894	58.0	59.2	0.60	3.71	774	2,114	1,444	1,373
H	228,654	250,188	41,274	34,340	14,568	7,568	2,089	655	53.5	54.6	0.51	4.03	774	2,381	1,578	1,467
I	211,089	208,956	40,153	36,029	11,827	8,196	2,391	584	57.6	55.4	0.53	3.46	773	2,270	1,521	1,458
mean	222,393 *	214,545 *	37,945 †	33,823 †	14,040 ‡	7,118 ‡	2,255 §	661 §	56.9 #	56.8 #	0.68	3.57	894	2,070	1,482	1,408
SD	12,320	18,512	3,821	3,202	1,860	842	193	125	1.7	1.8	0.17	0.38	130	228	63	53

Total numbers of cones automatically quantified in retinal wholemounts from experimental rats whose left eye had been exposed to Light Emitting Diode-induced phototoxicity (LIP) and were analysed seven days later. The numbers of cones within predetermined fixed-size circular areas (PCA) were also automatically quantified. The total areas of the retinas (Area of retina) as well as the areas lacking L- or S-cones (Area of damaged region) were measured over flat-mounted retinas. The maximum diameters (Diameter length of damage) of the areas lacking L- or S-cones were measured over flat-mounted retinas and also the length of ONL lacking cell nuclei was measured *in vivo* with OCT. Statistical analysis: *T-test p = 0.369; †T-test p = 0.049; ‡T-test p≤0.001; §T-test p≤0.001; # When compared with the areas of naïve retinas (see Table 2) there were no significant differences (ANOVA p = 0.974).

**Table 2:** Quantitative analysis of naive retinas.

Rat	Total number of cones in retinal wholemounts		Area of	Density in the whole retina (cones/mm^2^)	
	L-cones	S-cones	retina (mm^2^)	L-cones	S-cones
A	236,789	38,579	58.3	4064	662
A'	225,986	39,850	58.0	3896	687
B	228,564	40,297	56.8	4021	709
B'	216,661	37,958	55.0	3940	690
mean	227,000	39,171	57.0 #	3980	687
SD	8,290	1,088	1.5	76	19

Total numbers of cones automatically quantified in retinal wholemounts from naïve rats. The total areas of the retinas (Area of retina) were measured over flat-mounted retinas. The densities of L- and S-cones are provided. # When compared with the areas of the retinas from experimental animals (see Table 1) there were no significant differences (ANOVA p = 0.974).

**Figure 6:**
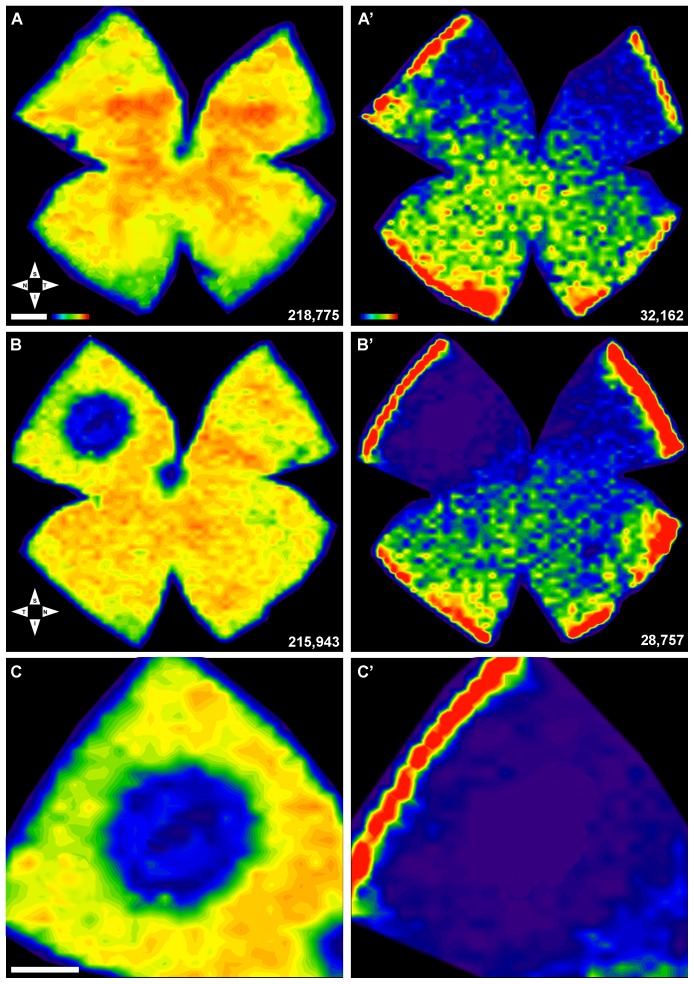
Topography of L- and S-cones in control and damaged retinas. Isodensity maps illustrating the topological distribution of L- (A,D,C) and S- (A′,B′,C′) cones in right control (unexposed) (A,A′) and the left (light exposed) (B,B′) retinas from a representative retina (same as shown in Fig.5) seven days after LIP. The maps are filled contour plots generated by assigning to each frame a color code according to its cone density value within a color-scale range from 0 (purple) to 6,500 or more (red) L-cones/mm^2^, or to 1,300 or more (red) S-cones/mm^2^. A,A′. In the control retina, L- (A) and S-cones (A′) are normally distributed throughout the retina, with highest concentrations of L-cones along the naso-temporal axis in the dorsal retina with maximum values in the superotemporal quadrant, while highest S-cones densities appear in the retinal rims and in the inferotemporal quadrant. B,B′. In contrast, the isodensity map of the light exposed retina (same retina illustrated in Figs. 5B,B′), demonstrates a small circular region with reduced densities of L- (B) and S-cones (B′) in the superotemporal quadrant at approximately 3.4 mm from the optic disc. Note that the area is larger for the S- (B′) than for the L-(A′) opsin. C,C′ magnifications of the superior-temporal quadrant shown in B and B′, respectively to illustrate the region lacking immunostaining of cone outer segments. Bottom right of each map: A,A′,B,B′. Total number of cones counted in that retina. S, superior; T, temporal; I, inferior; N, nasal. Bar: 1 mm.

Total numbers of L- or S-cones in naïve (n = 4) and fellow-right (n = 7) retinas of light exposed rats were 227,000±8,290 or 39,171±1,088 and 222,393±12,320 or 37,945±3,821, respectively, and these are comparable to previously reported data [1]. The total numbers of L- or S-cones in the light-exposed retinas were slightly or significantly smaller, respectively, than in their right retinas (**Tables 1** **,** **3**, **Fig. 6**). Thus, in addition to focal loss, there was also diffuse loss of cones and this was more apparent for the S-cones as observed in the isodensity maps (**Fig. 6**).

**Table 3:** Densities of L- and S-cones in LIP damage retinas.

	Density in the whole retina (cones/mm^2^)				Ratio between damaged and	
Animals					total retinal area (%)	
	L-cones		S-cones		L-cones	S-cones
	Right retina	Left retina	Right retina	Left retina		
C	3712	3642	600	649	1.04	5.44
D	3763	3869	708	608	1.31	6.80
E	3683	3778	667	503	1.51	6.45
F	4188	3292	548	631	1.60	5.42
G	4104	3551	682	505	1.02	6.27
H	4277	4583	772	629	0.94	7.38
I	3663	3773	697	650	0.96	6.25
mean	3913 β	3784 β	668 Φ	596 Φ	1.20	6.28
SD	265	400	74	65	0.28	0.70

Densities of L-and S-cones in retinas from experimental rats seven days after Light Emitting Diode-induced phototoxicity (LIP) in the left eyes, as well as the ratio between damaged and total retinal area. Statistical analysis, T-test: β p = 0.491; Φ p = 0.039.

**Figure 7:**
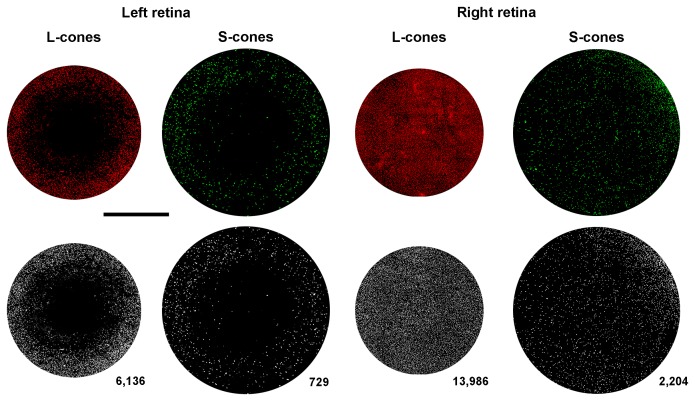
Automated quantification of L and S cones in predetermined-fixed-size circular areas of the retina. Top row. Composite micrographs of predetermined fixed-size circular areas (PCA) from one animal with LIP. Immunolabeled L- and S-cone outer segments in PCA from a representative left (light exposed) (same retina illustrated in Fig. 5B,B′) and right retina, 7 days after LIP. For each experimental retina and also within the corresponding region of the right-fellow retina, the numbers of L- or S-cones were counted within PCA of 3.14 mm^2^ or 5.3 mm^2^, respectively, superimposed upon the centre of the lesion. Bottom row. A custom-written macro allowed representation of every detected cone outer segment by a white dot within the PCA. Total numbers of L- or S-cones in these PCA was 7,118±842 or 661±125 for the light exposed retinas and 14,040±1,860 or 2,255±193 for control retinas, respectively (n = 7). Bar: 1 mm.

Total numbers of L or S cones in the predetermined fixed-size circular areas (PCA) analysed were 7,118±842 or 661±125 in the LED exposed retinas (n = 7) and 14,040±1,860 or 2,255±193 in the contralateral retinas (n = 7), respectively (**Table 1,** **Fig. 7**). Thus LIP results within the PCA measured, in the loss of approximately 49% or 71% of the L- or S-cone population, respectively.

### The loss of cones appears permanent and does not progress with time

#### Oriented Cross-sections analysis

**Figure 8:**
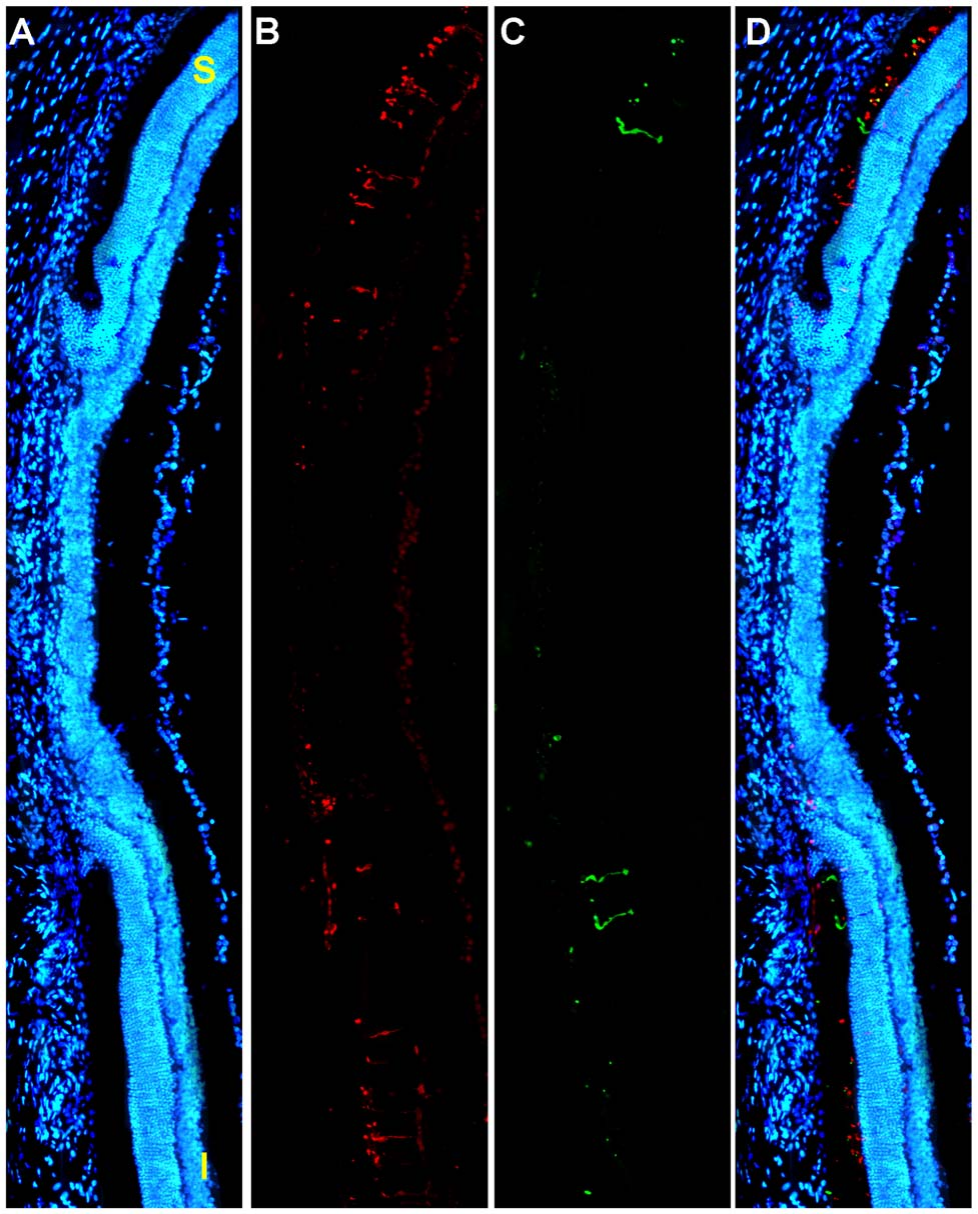
Radial parasagittal section of the retina illustrates focal damage. A–D. LIP results in focal damage to the outer retina. A. Photomontage of a representative parasagittal oriented radial section spanning the phototoxic lesion stained with DAPI (blue signal) to identify all cell nuclei, from the left retina of an adult albino rat 7 days after LIP. The section was also stained for S- and L-opsins. Note the focal region of damage that shows an absence of the outer nuclear and outer segment layers and results in massive loss of photoreceptors. B,C. L-opsin (red signal) (B) and S-opsin (green signal) (C). Outside the focal damage there are both L- and S-cones, while within the focal damaged region there is only some residual S- or L-immunoreactivity present in the outer segment layer of the retina. D. Couple images with nuclear staining DAPI (blue). S, superior. I, inferior. Bar: 500 µm.

Retinal cross-sections from fellow-right eyes had a normal appearance [1] as did also cross-sections from light-exposed retinas. However, upon closer inspection of the latter, a small region of damage could be found in the superotemporal retina. Within this region it was difficult to identify cell DAPI stained nuclei within the outer nuclear layer, although some residual L- or S-immunoreactivity was present in the outer segment layer of the retina. The damage, which extended for approximately 1,500 µms, had an abrupt start on both, superior and inferior limits, with an almost complete lack of cell nuclei (**Fig. 8**). These results document that within the light-damaged region of the retina there is massive loss of both rods and cone-photoreceptors.

#### Wholemounts analysed 7 or 30 days after LIP do not show progression of damage

**Figure 9:**
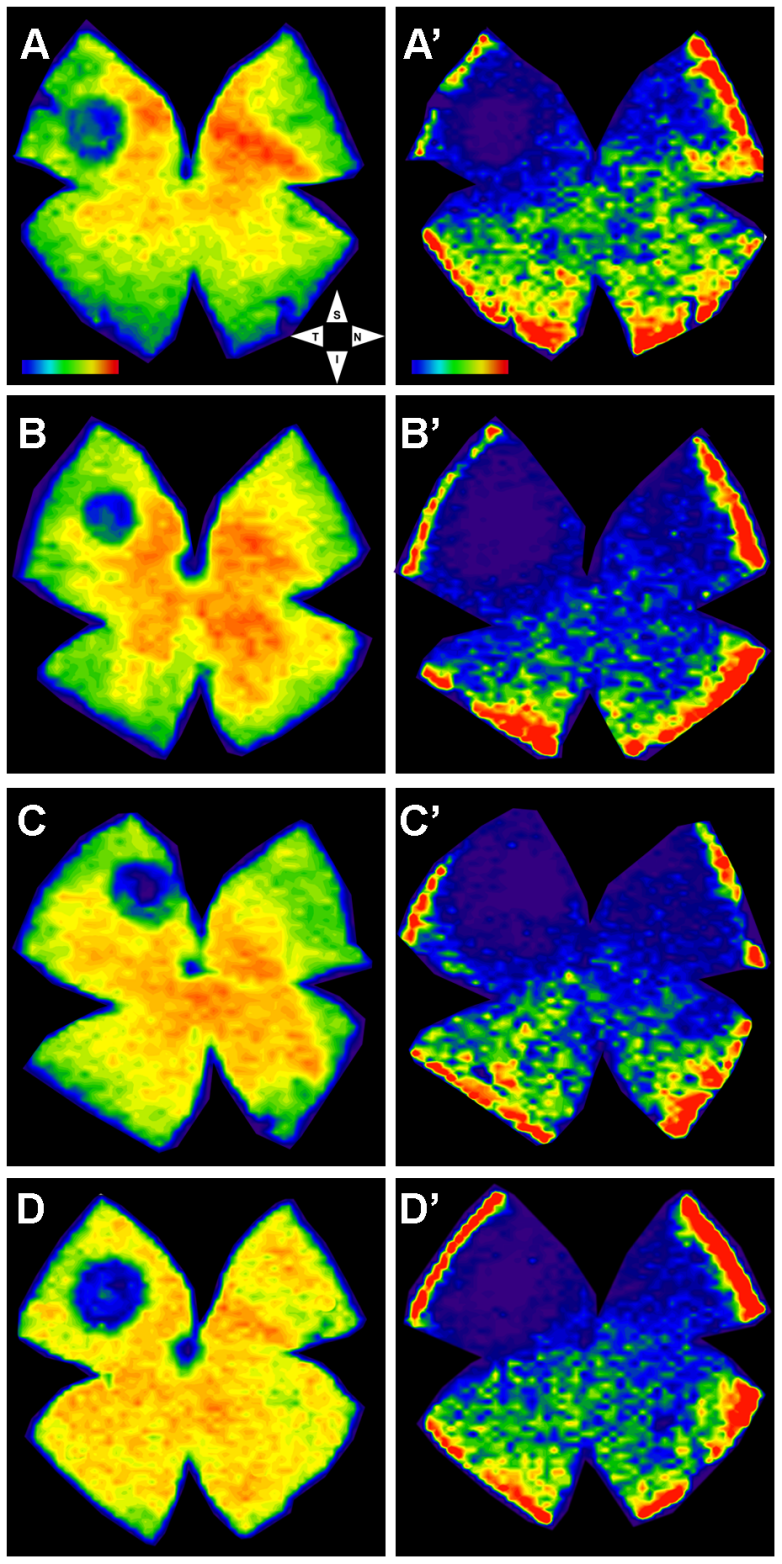
LIP results in consistent damage located in the superotemporal quadrant. A–D, A′-D′. Isodensity maps of four (A–D) representative left retinas, seven (A,B) or 30 (C,D) days after LIP. Isodensity maps were represented as a filled contour plot generated by assigning to each one of the subdivisions of each individual frame a color code according to its cone density value within a color-scale range from 0 (purple) to 6,500 or higher L-cones/mm^2^ (red) or to 1,300 or higher S-cones/mm^2^ (red). Note the presence in the left exposed retinas of a small focal lesion lacking L- (A-D) or S- (A′-D′) cone immunoreactivity that was consistently located in the superotemporal quadrant and appeared smaller for the L-(A–D) than for the S- (A′-D′) cones. S, superior; T, temporal; I, inferior; N, nasal. Bar: 1 mm.

To explore if the total number of missing cones or if the area of damage increased with longer survival intervals, we have compared two groups of rats examined 7 or 30 days after LIP. As described above, LIP resulted in a small circular region of damage consistently situated in the superotemporal quadrant, which was larger for S- than for the L-cones (**Fig. 9**). Total number of L- or S-cones, counted automatically within PCA of the left experimental retinas, were 7,383±1,047 (n = 8) or 580±211 (n = 8) and 7,711±1,245 (n = 8) or 627±71 (n = 8), at 7 and 30 days, respectively (**Table 4**), and these were comparable. The areas lacking L- or S-opsin immunoreactivity at 7 and 30 days were also comparable (**Table 4**). Thus, altogether these results suggest that neither the region with focal lesion nor the number of lost cones progress further between 7 and 30 days after LIP.

**Table 4:** Comparative analysis of retinal damage 7 and 30 days after LIP.

	7 days after LIP					
Animal	L-cones			S-cones		
	Right Retina	Left Retina	Damaged area (mm^2^)	Right Retina	Left Retina	Damaged area (mm^2^)
**A**	15,670	6,925	0.76	2,653	753	3.01
**B**	15,891	8,698	0.71	2,068	426	4.03
**C**	14,529	7,691	0.76	2,289	458	3.73
**D**	12,509	7,463	0.58	2,053	448	3.06
**E**	11,136	6,681	0.88	2,144	293	2.87
**F**	12,967	9,024	0.61	2,295	939	4.03
**G**	12,787	6,098	0.62	1,973	649	3.46
**H**	15,330	6,489	0.86	2,280	678	2.87
**mean**	**13,852**	7,384 *	**0.72 †**	**2,219**	**580 ‡**	**3.38 §**
**SD**	**1,740**	**1,047**	**0.11**	**214**	**211**	**0.50**

	30 days after LIP					
Animal	L-cones			S-cones		
	Right Retina	Left Retina	Damaged area (mm^2^)	Right Retina	Left Retina	Damaged area (mm^2^)
**A**	10,563	9,289	0.46	2,138	658	2.80
**B**	16,234	8,569	0.84	2,398	589	3.92
**C**	12,083	6,025	0.65	2,756	620	3.33
**D**	11,853	7,423	0.55	2,089	501	3.62
**E**	15,239	7,989	0.61	2,464	589	3.84
**F**	10,230	6,034	0.80	2,376	674	3.82
**G**	15,389	9,007	0.63	1,879	645	2.86
**H**	15,802	7,349	0.67	2,409	742	2.80
**mean**	**13,424**	7,711 *	**0.65 †**	**2,314**	**627 ‡**	**3.37 §**
**SD**	**2,489**	**1,245**	**0.12**	**270**	**71**	**0.49**

Comparative analysis of retinal damage at 7 or 30 days after Light Emitting Diode-induced phototoxicity (LIP). The number of L- or S-cones quantified in the predetermined fixed-size circular areas PCA, as well as the area lacking L- or S-cones (Damaged area) measured over flat-mounted retinas reveals that neither the area of the focal lesion nor the number of lost cones progress further between 7 and 30 days after LIP. Statistical analysis, T-test: * p = 0.579; † p = 0.253; ‡ p = 0.562; § p = 0.973.

### Topical brimonidine and Intravitreal BDNF, bFGF or PEDF prevent cone photoreceptor loss

**Figure 10:**
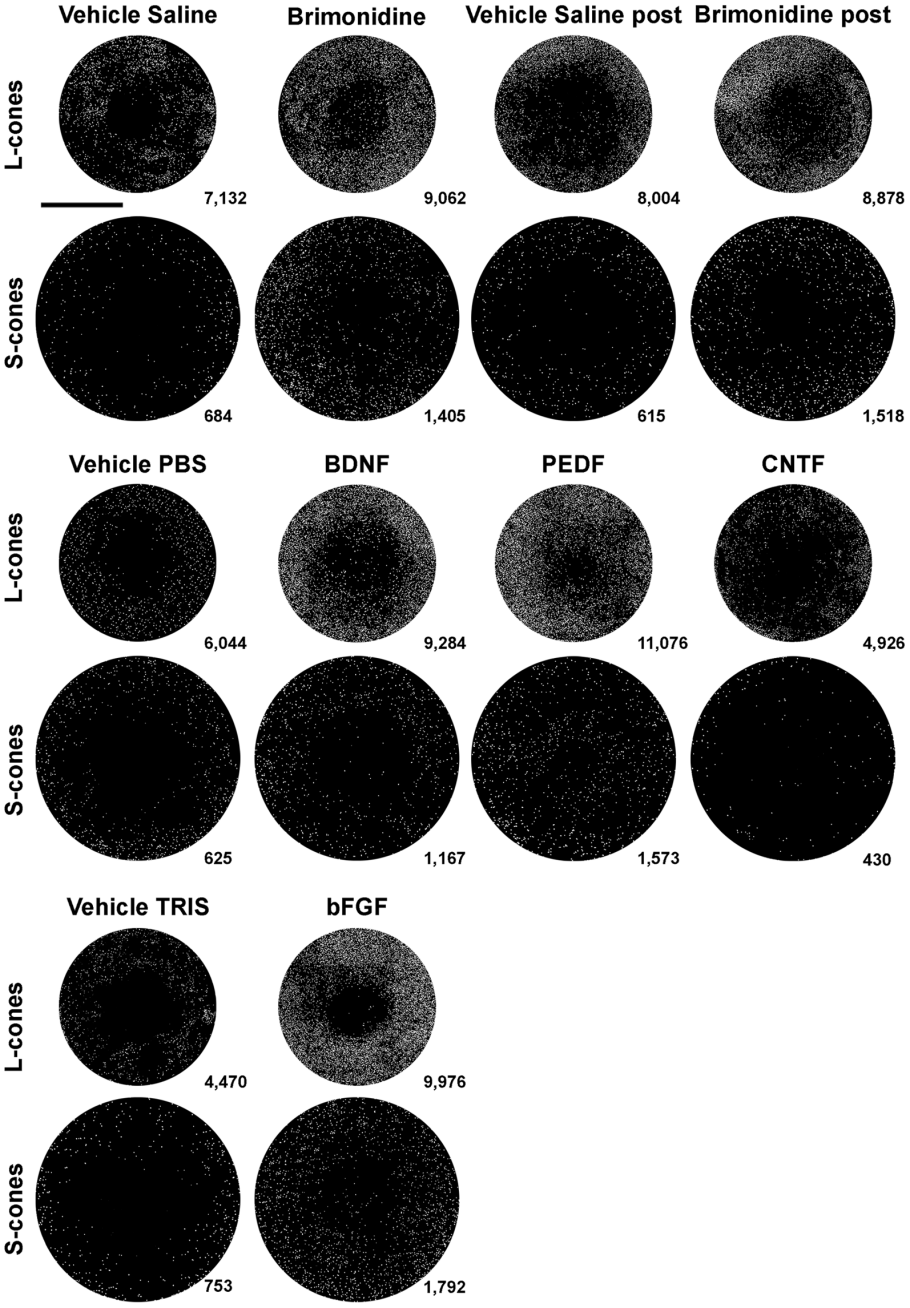
Cone survival in the predetermined fixed-size circular areas examined after treatment with topical Brimonidine or intravitreal BDNF, PEDF, bFGF or CNTF. Representative examples of the predetermined-fixed-size circular areas (PCA) analysed in the experimental eyes 7 days after blue-light emitting diode induced phototoxiticy (exposure parameters were 10 secs and 200 Lux) and treatment with topical Brimonidine (1%) or intravitreal BDNF (5 µg), PEDF (2 µg), CNTF (0.4 µg) or bFGF (1 µg), and the corresponding vehicle solutions. For each experimental and its fellow retina, a PCA (3.14 mm^2^ for L- and 5.3 mm^2^ for S-cones) was superimposed on the centre of the lesion and L- or S-cones were counted. Every cone is represented by a white dot. Lower bottom of each area shows the total number of cones counted. Note that the numbers of cones are greater in the BMD, BDNF, PEDF, bFGF treated group than in the corresponding Vehicle-treated groups, but not for the CNTF-treated group.

**Figure 11:**
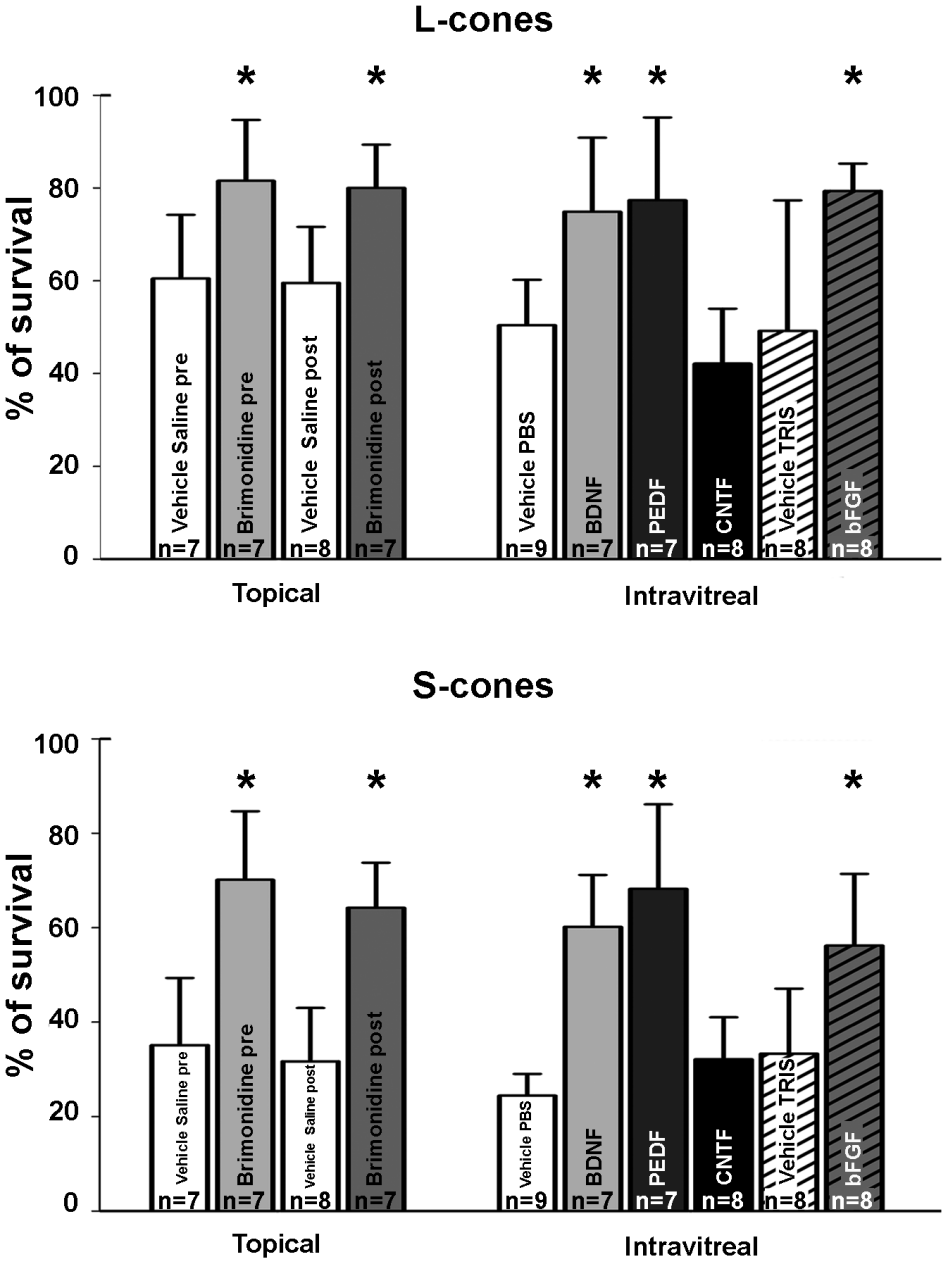
Histogram showing cone-survival (%) after different treatments. Histograms showing in percentages (light-exposed vs fellow) cone-photoreceptor survival 7 days after LIP and treatment with topical Brimonidine (1%) or intravitreal BDNF (5 µg), PEDF (2 µg), CNTF (0.4 µg) or bFGF (1 µg), and the corresponding vehicle solutions, measured within predetermined fixed size circular areas (3.14 mm^2^ for the L cones and 5.3 mm^2^ for the S cones). Histogram shows L-(top) and S-(bottom) cone survival and neuroprotection afforded by treatment with topical BMD, or intravitreal BDNF, PEDF or bFGF, but not CNTF or vehicle solutions. * p<0.05 (T-test).

The effects of topical BMD (1%) administered before or right after LIP, and the effects of BDNF (5 µg), PEDF (2 µg), CNTF (0.4 µg) or bFGF (1 µg) intravitreally injected after LIP were examined in wholemounts at 7 d. Total numbers of L or S cones counted automatically within PCA in experimental as well as in corresponding regions of their fellow retinas are detailed in **Table 5**, and a representative example from each group is shown in **Figure 10**. These results demonstrate that although intravitreal injection of CNTF (0.4 µg) does not provide significant neuroprotection, BDNF (5 µg), PEDF (2 µg) or bFGF (1 µg) are effective neuroprotectors against focal phototoxicity-induced cone degeneration, as it is also topically administered BMD (1%) (both pre- or post-LIP) (**Fig. 11** **, Table 5**).

**Table 5:** Neuroprotective effects of topical BMD or intravitreal BDNF, PEDF, CNTF or bFGF.

		L-cones		S-cones	
		Right retina	Left retina	Right retina	Left retina
	Vehicle Saline pre n = 7	12,509±1,111	7,463±1,256 *	2,425±538	812±299 †
Topical administration	Brimonidine pre n = 7	11,136±1,303	9,024±1,405 *	2,268±406	1,578±361 †
	Vehicle Saline post n = 8	13,273±1,640	7,775±1,038 ‡	2,195±347	689±238 §
	Brimonidine post n = 7	11,292±1,486	9,007±1,453 ‡	2,308±628	1,444±629 §
	Vehicle PBS n = 9	12,951±2,733	6,716±2,526 π¥ω	2,361±520	662±235 #Φδ
	BDNF n = 7	12,787±2,938	9,710±3,725 π	2,051±728	1,229±509 #
	PEDF n = 7	12,551±1,797	9,463±1,241 ¥	2,500±469	1,685±390 Φ
Intravitreal administration	CNTF n = 8	13,129±2,671	5,522±3,037 ω	2,386±288	709±204 δ
	Vehicle TRIS n = 8	13,532±1,743	6,617±3,614 ж	2,442±483	762±257 ю
	bFGF n = 8	13,438±2,175	10,662±1,830 ж	2,544±351	1,414±398 ю

Comparative analysis between groups treated with neuroprotective agents or corresponding vehicles indicates that there was a significant greater number of cones in each treatment with respect to the vehicle treated group except for the CNTF treated group. Statistical analysis, T-test: * p = 0.047; † p≤0.001; ‡ p = 0.049; § p≤0.001; π p = 0.035; # p = 0.004; ¥ p = 0.02; Φ p≤0.001; ω p = 0.305; δ p = 0.66; ж p = 0.013; ю p = 0.002.

## Discussion

In the present study we have characterized for the first time, *in vivo* with SD-OCT as well as *ex vivo* with fluorescence microscopy, the effects of a small phototoxic retinal lesion on the survival of L- and S-cones in the adult albino rat retina. Using a blue (400 nm) LED, molecular markers to identify L- and S-cones as well as recently developed technology to image their distribution and to count automatically the population of L- or S-cones, we document that blue-LED-induced phototoxicity (LIP) is a reproducible and quantifiable model to study light-induced cone degeneration. We also show that SD-OCT is a reliable technique to examine *in vivo* the effects of LIP. Moreover, intravitreal administration of BDNF, PEDF or bFGF, or topical administration of BMD results in significant cone neuroprotection against phototoxicity-induced degeneration.

For the present experiments albino rats were chosen instead of pigmented because of their greatest sensibility to light-induced retinal damage. Previous studies from our group and others have documented that light damage to the retina depends in addition to light source and duration of exposure, on eye pigmentation [7], [74]–[76] as well as light wavelength [77], [78]. Furthermore, it has been documented that in contrast to pigmented rats [7], [12], [13], [74], in albino rats, pupil dilation is not necessary for the damage to occur [13]. However, in albino rats, mydriasis increases the phototoxic damage [13].

Our *in vivo* image analysis using SD-OCT showed that retinal damage induced by LED was circumscribed to a circular region that decreased from 1 to 7 days after LIP. Such a well-defined circle of damage might be due to a combination of the source of light by the LED and the rat's optics that concentrate irradiation into a well-defined circular area of the retina, as has been shown for other specific sources of light [79], [80]. It is possible that the decrease in the main diameter of the region affected that occurs from day 1 to day 7 relates to the presence of a transient inflammatory reaction at the peripheral border of the lesion which would resolve in the following days. Indeed, the observation that the areas lacking L- or S-opsin immunoreactivity at 7 and 30 days were comparable indicates that such a diminution in size is completed by one week after LIP. We cannot discard however that such a decrease in the size of the lesion is due to the migration of surrounding photoreceptors into the lesion, as has been shown to occur in adult pigmented rats up to three weeks after focal loss of photoreceptors induced by 380 nm light [79].

The distribution of L- and S-cones in the albino rat retina is not homogeneous, with marked regional variations in their densities. Of particular interest is that L-cones densities parallel highest RGC densities, whereas highest S-cone densities are found within the retinal rim and inferotemporal quadrant, where the lowest L-cone densities appear [1], [6], [73]. Thus, although the rat retina does not have a macula or fovea, its cone to rod ratio is similar to that of the human retina and more importantly it has a defined retinal specialized area in the temporal tip of the visual streak with maximum L-cone densities that may be used to study cone-photoreceptor injury. Our results show in the light-exposed retinas a circular region with focal loss of L- and S-opsin immunoreactivity, consistently located in the highest L-cone density region of the retina. In addition to this focal lesion, total counts in retinal wholemounts indicate that there was also a diffuse loss of cones within the retina, but this was more apparent and only statistically significant for the S-cones. The possibility that a small fraction of the light provided by the LED is scattered throughout the retina and absorbed elsewhere on the retina could explain the diffuse cone-photoreceptor cell loss. In previous experiments in which retinal damage was induced by diffuse light in rats, photoreceptor degeneration was maximal in an arciform region of the dorsal retina [12], [23], an area that has been termed the “photosensitive area” of the rat retina, but there was also diffuse loss of photoreceptors throughout the retina [12], [12], [15], [68], [74], [81], [82,83]. The inter-animal variability in total number of cones [1], [2] as well as the fact that the circular region with diminished numbers of cones represented only approximately 1.3% of the entire retinal area (Table 3), might explain why the overall total number of L-cones was not modified significantly. Previous studies have indicated that blue light is highly phototoxic to photoreceptors, and that specific light sources of light could result in focal lesions that did not progress with time [79], [80]. However, to the best of our knowledge, the outcome of the L- or S-cone population had not been specifically investigated before, mainly because the specific tools presently used were not available [84] and cones only represent, as a whole, approximately 1% of the photoreceptor population [64], [65].

Our present studies have relied on the identification of cone-outer segments with immunohistochemistry, using specific antibodies against specific L- or S-cone opsins. It has been shown that the expression of specific opsin proteins within cone-photoreceptors may be modified and even disappear after retinal injury [42], and this may lead to misinterpretation of cone-photoreceptor cell survival. However, both our OCT analysis *in vivo* as well as our *ex vivo* cross-section analysis indicate that in the regions lacking cone-photoreceptors there were no other cell nuclei, thus further confirming the absence of both cones and rods. In our studies LIP results in massive focal loss of cone and rod photoreceptors and as previously suggested [79], [80], it is likely that blue light affects RPE cells and induces the loss of both rods and cones (see below). However, the molecular and cellular mechanisms responsible for such a massive loss of photoreceptors remain a matter for future studies. Here we have chosen a blue-light emitting diode as a source of light irradiation because it induces particularly harmful phototoxicity [11], [85], [86]. Photochemical damage may involve the formation of free radicals and thus oxidative stress following excessive photoreceptor photopigment activation [7] or the effect of short wave length linked to chemical changes in lipofuscin [9]. In our studies, the blue-LED provided 400 nm light, and this has been shown to induce retinal pigment epithelium photoxicity [87], [88] mediated by lipofuscin [10]. Indeed, cryostat cross-sections stained with methylene blue showed that the retinal pigment eptithelium is largely affected in the focal area of retinal lesion. Moreover, the specific toxicity of short wave-lengths has been related to the chromophore present in lipofuscin (the A2E, N-retinylidine-N-retinylethanolamine) [10] and this has also been shown in vitro [89].

A large number of experimental studies have established the neuroprotective effects of alpha-2 selective agonists against a variety of retinal injury models [90]–[93] including transient ischemia-induced RGC loss [94]–[97], ocular hypertension induced retinal damage [98] and light-induced photoreceptor degeneration [21]. However, a neuroprotective effect on L- and S-cones against LIP had not been shown before, and it is possible that its mechanism of action is related to upregulation of trophic factors that contribute to neuronal survival [24], [99] or to the activation of extracellular signal-activated kinases [100] or to the transactivation of epidermal growth factors [101] through Müller cells activation.

Trophic factors are endogenous substances with critical functions during neuronal development and survival. In general their functions are related to promote proliferation, regeneration, maturation and/or survival of neurons [20]. For example, intravitreal administration of neurotrophins such as BFNF, neurotrophin-4 and CNTF has been shown to prevent ON injury-induced RGC death [71], [102]–[104]. Exogenous administration of these substances has been shown to have an important role in treating degenerative retinal diseases due to an increased expression in the target cells [105]. Previous studies on the effects of intravitreal administration of trophic factors including BDNF [25], [26], [28], [29], [34], [35], PEDF [22], [106], [107], bFGF [21], [33], [108], or CNTF [29], [108]–[112] have shown prevention of phototoxic-induced neurodegeneration of photoreceptors [20]. Overall, our results are in agreement with these studies, but differ in that we have studied and documented selectively the neuroprotection of L- and S-cones against LIP and also in that in our studies CNTF did not confer neuroprotection. The mechanism of action of these trophic factors to prevent light-induced phototoxicity is probably direct and indirect through Müller cells [113], [114] or enhancing rod-photoreceptor survival [115], and it is possible that at the doses used in our experiments CNTF failed to produce neuroprotection. Indeed, in the present studies we have used an intravitreal injection of 0.4 µg of CNTF, a dose that is several orders of magnitude greater than that reported to afford photoreceptor neuroprotection [29], and other studies have shown that high doses of intravitreal CNTF have functional and morphological deleterious effects on adult rat photoreceptors [32], [111].

In summary, a detailed quantitative study taking into account the whole population of cone-photoreceptors, as well as its topological representation following light-induced damage had not been reported before. The present studies document for the first time that blue LED-induced phototoxicity is a reproducible and quantifiable model of focal cone degeneration induced in the rat visual streak to study light-induced cone degeneration, which might be assessed in vivo with SD-OCT. This is even more relevant if one takes into account that, on average, approximately 99% of the rat photoreceptors are rods while cone-photoreceptors account for only 1% [64], [65]. Intravitreal administration of BDNF, PEDF or bFGF but not CNTF, or topical administration of BMD results in significant cone neuroprotection against phototoxicity induced degeneration. It is anticipated that the present studies may lay the foundations for further studies analyzing the effect of other strategies aimed to prevent light-induced cone degeneration.
